# Consistency requirements determine optimal noise correlations in neural populations

**DOI:** 10.1186/1471-2202-14-S1-F1

**Published:** 2013-07-08

**Authors:** Joel Zylberberg, Maxwell Turner, Yu Hu, Jon Cafaro, Greg Schwartz, Fred Rieke, Eric Shea-Brown

**Affiliations:** 1 Department of Applied Mathematics, University of Washington, Seattle, WA 98195, USA; 2 Department of Physiology and Biophysics, University of Washington, Seattle, WA 98195, USA; 3 Howard Hughes Medical Institute, University of Washington, Seattle, WA 98195, USA

## 

A key challenge in population coding is to understand the role of correlations between the activities of different neurons. While the existence of correlations in primary visual cortex (for example), is somewhat controversial, retina presents a relatively clean story, with many studies observing that correlations exist and are important in shaping the population activity distribution. Given the retina's role in conveying visual information to the brain, and the relative clarity of the experimental data, retina offers a unique opportunity to study how correlations affect neural function. This question has received much attention, and previous work emphasizes that we must distinguish between two important types of correlations. First, there are the signal correlations, which describe how the mean (averaged over trials of the same stimulus) responses of two cells co-vary as the stimulus is changed. The noise correlations, on the other hand, describe how two neurons' responses co-vary over the repeat trials of the same stimulus. How do signal- and noise- correlations inter-relate with respect to population coding? Several theoretical principles have emerged. For example, Averbeck et al. [1] showed that, for optimal discriminability of two different stimuli, a pair of neurons should have opposite signs for their signal- and noise- correlations: positive signal correlations demand negative noise correlations, and vice versa. This "opponent signal and noise correlations" situation yields better discriminability than occurs with uncorrelated noise, and these results do extend to larger populations. For heterogeneous populations, subsequent works indicates that the situation is more nuanced.

To experimentally test these theoretical ideas, we measured the noise correlations for a population of direction selective retinal ganglion cells with different signal correlations for different pairs, and observed that, regardless of the signal correlations (positive for some cell pairs, and negative for others), all neural pairs had small positive noise correlations. This is in contrast with the notion of opponent signal and noise correlations. To understand this discrepancy, we created a simple mathematical model of our experimental system, in which the overlap between two neurons' tuning curves dictates their signal correlations; the signal correlations differed between cell pairs, and belong to the set {0, 1,-1}. The noise correlations are the independent variable for our numerical experiments. Note that our model population has 8 (>2) neurons in it. In our model, optimal coding performance (measured, for example, using linear Fisher information) occurs when the noise covariance matrices lie on a boundary of the space of allowed covariances. Recall that only positive definite covariance (and correlation) matrices are possible, which means that correlations between pairs need to be consistent across the population; this requirement shapes the boundary. For example, if neurons A and B have a perfect noise correlation (ρ_AB _= 1), and so do neurons B and C (ρ_BC _= 1) then neurons A and C must also have a perfect noise correlation (ρ_AC_ = 1). One cannot choose (ρ_AB_, ρ_BC_, ρ_AC _) = (1,1,-1), for example, and pair-by-pair arguments about what the noise correlations should be will necessarily miss this restriction. We have further proven mathematically that, regardless of the encoder details, the optimal encoder, using either OLE performance, or linear Fisher information as a metric, must lie on a boundary of the space of allowed noise covariance matrices. The small all-positive noise correlations we observed yielded near-optimal performance in our model population. Considering one pair at a time, it might be better to choose negative noise correlations for cell pairs with positive signal correlations and vice versa, but such choices may be impossible, due to the requirement that the correlations be consistent across the population. Since the optimal solutions must lie on the boundary of the allowed space of correlation matrices, the consistency requirement is a critical factor in determining the noise correlation structure that optimizes population coding.
